# *In Vitro* Differentiation of Neural-Like Cells from Human Embryonic Stem Cells by A Combination of Dorsomorphin, XAV939, and A8301

**DOI:** 10.22074/cellj.2018.4232

**Published:** 2017-11-04

**Authors:** Zahra Valizadeh-Arshad, Ebrahim Shahbazi, Shiva Hashemizadeh, Azadeh Moradmand, Meyssam Jangkhah, Sahar Kiani

**Affiliations:** 1 Department of Stem Cells and Developmental Biology, Cell Science Research Center, Royan Institute for Stem Cell Biology and Technology, ACECR, Tehran, Iran; 2 Department of Developmental Biology, University of Science and Culture, ACECR, Tehran, Iran; 3 Department of Embryology, Reproductive Biomedicine Research Center, Royan Institute for Reproductive Biomedicine, ACECR, Tehran, Iran

**Keywords:** Differentiation, Embryonic Stem Cells, Motor Neuron, Small Molecule, Whole-Cell Patch Clamp

## Abstract

**Objective:**

Motor neuron differentiation from human embryonic stem cells (hESCs) is a goal of regenerative medicine to provide cell therapy as treatments for diseases that damage motor neurons. Most protocols lack adequate efficiency in generating functional motor neurons. However, small molecules present a new approach to overcome this challenge. The aim of this research is to replace morphogen factors with a cocktail of efficient, affordable small molecules for effective, low cost motor neuron differentiation.

**Materials and Methods:**

In this experimental study, hESCs were differentiated into motor neuron by the application of a small molecule cocktail that consisted of dorsomorphin, A8301, and XAV939. During the differentiation protocol, we selected five stages and assessed expressions of neural markers by real-time polymerase chain reaction (PCR), immunofluorescence staining, and flow cytometry. Motor neuron ion currents were determined by whole cell patch clamp recording.

**Results:**

Immunofluorescence staining and flow cytometry analysis of hESC-derived neural ectoderm (NE) indicated that they were positive for NESTIN (92.68%), PAX6 (64.40%), and SOX1 (82.11%) in a chemically defined adherent culture. The replated (hESC)-derived NE differentiated cells were positive for TUJ1, MAP2, HB9 and ISL1. We evaluated the gene expression levels with real-time reverse transcriptase-PCR at different stages of the differentiation protocol. Voltage gated channel currents of differentiated cells were examined by the whole-cell patch clamp technique. The hESC-derived motor neurons showed voltage gated delay rectifier K^+^, Na^+^ and Ca^2+^ inward currents.

**Conclusion:**

Our results indicated that hESC-derived neurons expressed the specific motor neuron markers specially HB9 and ISL1 but voltage clamp recording showed small ionic currents therefore it seems that voltage gated channel population were inadequate for firing action potentials.

## Introduction

Embryonic stem cells (ESCs) are pluripotent cells derived from pre-implantation blastocysts (1) that have the ability to self-renew and differentiate into all cell types within the body. Potential use of human ESCs (hESCs) in regenerative medicine depends on their directed differentiation of specific lineages. The effective method of differentiation is a critical need for direct differentiation of hESCs to specialized functional cell types (2). In the past two decades numerous protocols have been devised to differentiate hESCs into populations derived from specialized subtypes of neurons, including motor neurons among others of the neural lineage. In most of these protocols growth factors and small molecules were applied for direct differentiation. Small molecules for their reasonable price, good efficiency and costeffectiveness have widely used in novel approach of neural differentiation (3). Therefore, in the present research small molecules were employed for effective hESCs differentiation into motor-neurons. Small molecules are small chemical molecules with low molecular weight, selective approach and the ability to mimic cell signaling pathways. Transforming growth factor beta (TGFβ), bone morphogenetic protein (BMP), and Wnt signaling pathways inhibition promote differentiation of hESCs along the neuronal lineage (4, 5). According to previous research retinoic acid (RA) and sonic hedgehog (SHH) have been considered as the caudalized and ventralized factors for neural differentiation (6). Purmorphamine is a small molecule that can mimic SHH signaling pathway and ventralize neural precursors (7). A8301 is a selective inhibitor for the TGFβ signaling pathway whereas XAV939 is a beta-catenin-mediated transcription inhibitor of the selective Wnt pathway. The combination of A8301 and XAV939 drive neural tubes to be caudalized (5, 7). The purpose of this research is to replace morphogen factors with a cocktail of efficient, affordable small molecules for effective, low cost motor neuron differentiation.

## Materials and Methods

### Culture of human embryonic stem cells

In this research Royan H6 cell line was used that established and approved by Royan Institute ethics committee (8). hESCs colonies were maintained under feeder-free culture conditions at 37˚C, 5% CO_2_, and 95% humidity in hESC medium that consisted of Dulbeccos’ modiﬁed Eagles’/Hams’ F12 medium (DMEM/F12, Invitrogen), 20% knockout serum replacement (KOSR, Invitrogen), 1% nonessential amino acids (Invitrogen, USA), 2 mM L-glutamine (Invitrogen, USA), 1 mg/ ml insulin, 0.55 mg/ml transferrin, and 0.00067 mg/ml selenium (ITS, Invitrogen, USA), 100 mM β-mercaptoethanol (Sigma, USA), and 100 ng/ml basic ﬁbroblast growth factor (bFGF, Royan Institute, Iran). hESC colonies were enzymatically and mechanically passaged every 7 days on plates coated with a thin layer of Matrigel (1:30, Sigma Aldrich, USA) and their medium made a refresh daily.

### Neural differentiation of human embryonic stem cells

Neural differentiation was induced based on a previously published protocol with modifications and the addition of small molecules (9). This protocol was an optimized adherent culture without embryoid body formation. The differentiation protocol consisted of the following: stages: i. Neural induction by dorsomorphin, XAV939, A8301, RA, and bFGF (stages 1 and 2), ii. Suspension culture (stage 3), iii. Motor neuron differentiation from hESC-derived neural progenitor cells (NPs) NPs by purmorphamine and RA (Stage 4), and iv. Mature neural cells (stage 5).

Initially, in stage 1 hESCs were directed into neural ectoderm in induction medium that contained DMEM/ F12 medium, 5% KOSR, 2% N_2_ (Invitrogen, USA, including recombinant insulin, human transferrin, sodium selenite, putrescine and progesterone), 10 ng/ mL *basic fibroblast growth factor* (bFGF), 2 μM alltrans- RA (Sigma-Aldrich, USA), and small molecules 2.5 μM dorsomorphin (Sigma-Aldrich, USA), 2 μM A8301 (Sigma-Aldrich, USA) and XAV939 (0.1 μM) for 4 days. In stage 2 all small molecules were eliminated, with the exception of RA, and applied 25 ng/ml bFGF in the same induction medium for 14 days. At day 18, these structures were manually sectioned from the surrounding cells by a sterile pulled-glass pipette visualized under a phase-contrast microscope (×10, Olympus, Japan). The structures were cultured as a suspension in a bacterial plate in the same medium without bFGF and in the presence of RA (2 μM) and purmorphamine (1 μM) for 2 days. In stage 3, purmorphamine and retinoic acid administrated for motor neuron differentiation. After stage 3, neural tube-like structures (NTs) were landed on tissue culture plates coated with 5 mg/ml laminin (Sigma- Aldrich, USA) and 15 mg/ml poly-L-ornithine (PLO, Sigma-Aldrich, USA) for motor neuron differentiation consisted of neurobasal medium (USA) supplemented with 1% N_2_, 2% B27 (Invitrogen, USA), 2.5% KOSR, 200 μM ascorbic acid (Sigma Aldrich, USA), 2 μM RA, and 1 μM purmorphamine for 6 days (stage 4). In stage 5 the cells were exposed to lower concentration of purmorphamine (0.2 μM) in the same induction medium for more 6 days.

### Immunoﬂuorescence staining

The expressions of cytoplasmic and nuclear proteins were evaluated by Immunoﬂuorescence staining. The cells were fixed in 4% paraformaldehyde (Sigma-Aldrich, USA) for 1 hour, then subsequently permeabilized with 0.1% Triton, USA X-100 and blocked in 4% bovine serum albumin (BSA) with 10% goat serum in phosphate-buffered saline (PBS) for 45 minutes at room temperature and then primary antibody applied at 4˚C. After, the cells were washed and incubated with secondary antibodies conjugated with either ﬂuorescein isothiocyanate (FITC) or Texas red, as follows: goat anti-mouse IgG-Texas red, goat anti-rabbit IgG-Texas red, and mouse anti-goat IgG-FITC for 45 minutes at room temperature. The primary antibodies consisted of mouse IgG NESTIN, rabbit polyclonal IgG PAX6, and mouse IgG MAP2. Finally, the nuclei were stained with 1 mg/ml of 4, 6-diamidino-2-phenylindole (Sigma-Aldrich, USA) for 3 minutes at room temperature. Cells were washed in washing buffer that contained 50 μl Tween (0.05%) in PBS after every stage.

### Flow cytometric analysis

At first, cells were washed with PBS and dissociated with trypsin-ethylene diamine tetra acetic acid (EDTA, Sigma-Aldrich, USA). After determination of cell viability by trypan blue exclusion, the cells were ﬁxed in ethanol and acetone for 30 minutes. After washing, the cells were permeabilized and blocked with Triton X-100 (0.1%), 29 mg/ml EDTA, and 1 mg/ml BSA in 50 ml PBS for 30 minutes at 4˚C. Then, primary antibody was applied overnight at 4˚C. Then, 1-1.5×10^5^ cells counted per each sample. After washing, the cells were stained with secondary antibodies for 60 minutes at 4˚C. The isotype control contained only the secondary antibody. All experiments were repeated three times and the acquired data was analyzed with WinMDI2.9 software.

### RNA isolation and quantitative reverse transcriptionpolymerase chain reaction

Gene expression patterns were evaluated in the neural tube like structure (stage 2) and maturation stage (stage 5) in comparison with hESCs (stage 0). Total RNA extracted by the Trysol and DNA contamination removed by the RNase-free DNase kit (Fermentas, Thermo scientific, USA). cDNA synthesized by 1 μM of total RNA using the RevertAid-H Minus First Strand cDNA Synthesis kit (Fermentas, Thermo scientific, USA). The data from sample replicates was expressed as fold change (mean ± SEM), as determined by the ΔΔCT method.

### Electrophysiology

Whole cell patch clamp technique was used to study the function of ion channels. NT-like structures were cultured on a coverslip coated with PLO and laminin. Resting membrane potential was measured in the current clamp mode and inward or outward ion currents were recorded in voltage clamp mode at days 11 and 20 after replating the NT-like structures. All recordings were performed at room temperature (25˚C). Patch electrode (ﬁlament borosilicate glass, 1.5 mm outer diameter, Harvard apparatus) resistance was 3-5 MΩ and pulled by a horizontal puller (Sutter Instruments). The recorded signals were ampliﬁed and ﬁltered (2 KHz) using a Multiclamp 700B ampliﬁer (Axon Instruments, USA). Ampliﬁed signals were acquired at 10 kHz using a Digidata 1440 analogto- digital (A/D) board and pCLAMP™ 10 software (Axon Instruments, USA). The signals were analyzed by Clampﬁt10 software (Axon Instruments, USA) in the off-line mode. The extra-cellular solution consisted of: NaCl 140 mM, KCl 4.5 mM, CaCl_2_ 2 mM, MgCl_2_ 1 mM, 4-(2-hydroxyethyl)-1-piperazineethanesulfonic acid (HEPES) 10 mM and glucose 10 mM and pipette solution contained: KCl 140 mM, CaCl_2_ 2 mM, MgCl_2_ 2 mM, ethylene glycol-bis (β-aminoethyl ether)- N,N,N’,N’-tetraacetic acid (EGTA) 2 mM, and HEPES 10 mM.

For recording inwardly voltage gated channels and eliminating the effect of K^+^p currents K^+^-free solution was used with compounds consisted of: NaCl 160 mM, CaCl_2_ 2 mM, HEPES 10 mM, glucose 10 mM for extra-cellular solution and CsCl 130 mM, MgCl_2_ 2 mM, EGTA 10 mM, TEA-Cl, 20 mM, HEPES, 10 mM, and glucose, 10 mM for pipette solution. Also Barium chloride solution was applied for recording calcium inwardly voltage gated channels because Barium chloride can mimic the function of calcium and passed from calcium channels. The barium chloride extracellular solution contained: NaCl 140 mM, MgCl_2_ 2 mM, BaCl_2_ 10 mM, CaCl_2_ 2 mM, glucose 5 mM, TEACl 5 mM, and HEPES 10 mM. Its pipette solution contained: CsCl 130 mM, MgCl_2_ 2 mM, CaCl_2_ 0.5 mM, EGTA 5 mM, and HEPES 10 mM.

### Statistical analysis

Statistical analysis was performed with SPSS (version 16) and Graph-pad prism (version 5, Graphpad software). All of the data were analyzed by the students’ t test. Experiments were performed in three independent cultures. Data were presented as mean ± SEM. P<0.05 were considered signiﬁcant.

## Results

### Combination of small molecules effectively induced human embryonic stem cells into neural precursors

**Fig.1:**
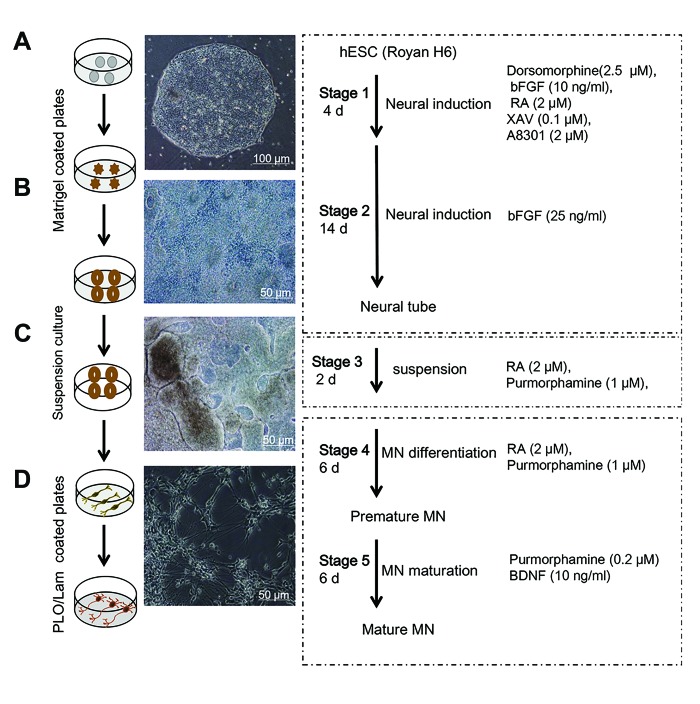
Schematic illustration of hESCs differentiation into motor neurons. A. Phase-contrast of hESC, B. Rosette structures, C. NT-like structures, and D. hESC-derived motor neurons. hESCs; Human embryonic stem cells, bFGF; Basic ﬁbroblast growth factor, RA; Retinoic acid, PLO/Lam; Poly-l-ornithine/laminin, BDNF; Brain-derived neurotrophic factor, and MN; Motor neuron.

**Fig.2:**
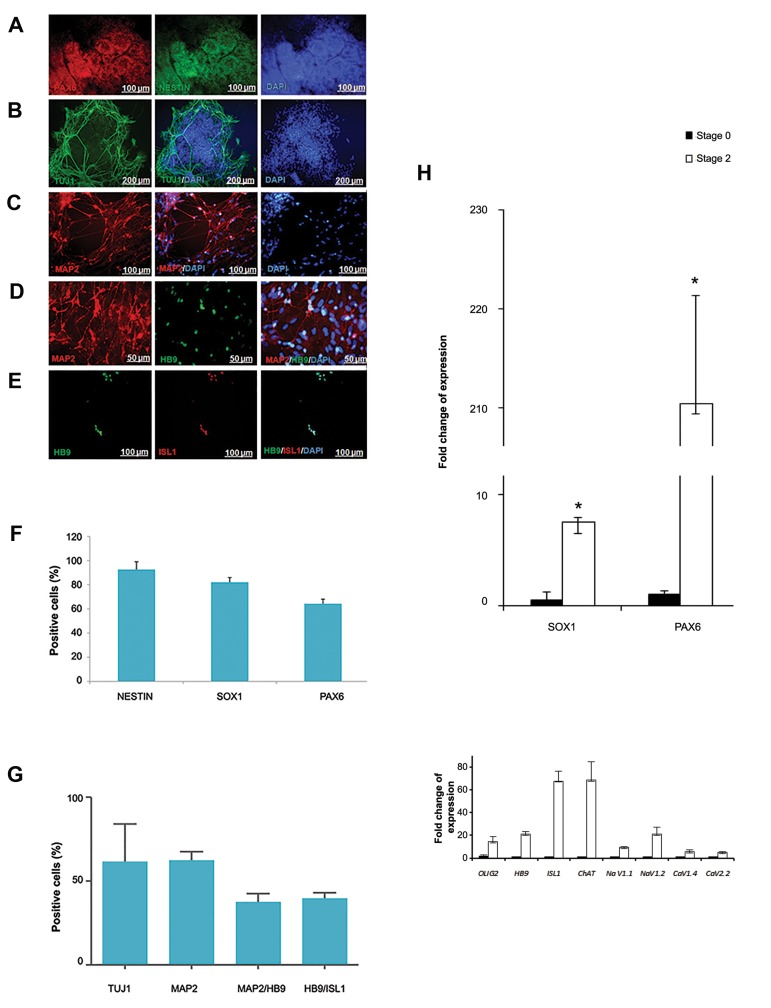
Characterization of human embryonic stem cells-derived neural cells. Immunoﬂuorescence staining showed that neural precursors (stage 2) expressed the main markers of these cells, A. PAX6, B. NESTIN, C. PAX6 and NESTIN, D. Blue color represents nuclei counterstained with DAPI, E. Flow cytometry analysis shows expression of NESTIN, SOX1 and PAX6 in neural precursors (stage 2), F. Quantiﬁcation of immunoﬂuorescence staining for mature neural and motor neuron markers, G. Immunoﬂuorescent microscopy of differentiated cells showed differentiated neuronal cells with long processes positive for mature neural markers (stage 5) TUJ1, and H. MAP2, Double staining of the differentiated cells showed expression of MN markers (stage 5).

Morphological changes in cellular plates and columnar cells initiated and neural differentiation observed after first 6 days. Subsequently, neural rosette structures formed 12 days after neural induction. Gradually, NTlike structures formed on day 18 with three dimensional structures and lumen (Fig .1). Immunofluorescent staining and Flow cytometry analysis showed these structures expressed neural progenitor proteins NESTIN (92.68 ± 6.33%), PAX6 (64.40 ± 3.46%) and SOX1 (82.11 ± 3.84%) (Fig .2).

### Motor neuron differentiation from human embryonic stem cells-derived neural precursors

Phase-contrast microscopy results represented neuronal-like cells appeared after neural tube structures plating on PLO/Laminin coated culture dishes (stage 4) and the cells neuritis wildly spread during next days. These cells expressed neural markers such as TUJ1 and MAP2 in stage 4. Then, 11 days after replating motor neuron markers (HB9 and ISL1) were expressed too. Gene expression results indicated that expressions of *OLIG2, HB9, ISL1* and *ChAT* increased in 11 days after replating (stage 5) compared to hESC (stage 0) (Fig .2).

### Human embryonic stem cells-derived motor neurons displayed neuron-like currents

**Fig.3:**
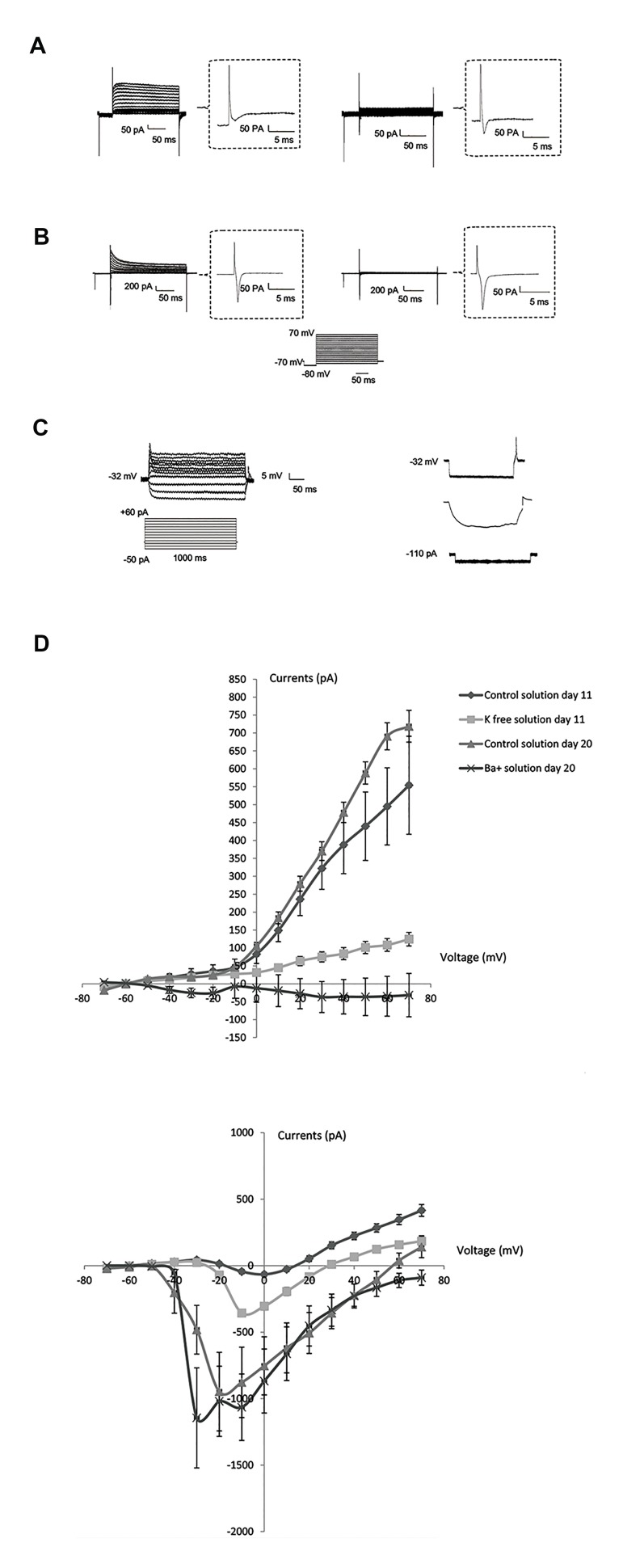
Electrophysiological properties in differentiated neural cells (stage 5). A. Current clamp recordings in differentiated motor neurons, B. Rebound action potential was detected in -110 pA in the presence of a control solution and its slope decreased in the presence of the BaCl solution at the same current, C. Voltage clamp recording shown on day 11 in the presence of the control solution, and D. Voltage clamp recording shown on day 11 in the presence of the K+-free solution.

Whole cell patch clamp recording data showed the resting membrane potential (RMP) was around -9.15 ± 0.74 mV (n=20) and -20.73 ± 3.87 mV (n=25) and input resistance was 1.38 MΩ and 1.01 MΩ on day 11^th^ and 20^th^ respectively. In a current clamp mode (injected current-50 pA to +60 pA) only single action potential was recorded in 20^th^ day and interestingly, after injection of hyper-polarization current (injected negative currents from-110 pA) rebound action potentials were recorded at day 20 that decreased in barium chloride solution (Fig .3). For recording currents from voltage gated ion channels voltage steps applied for 500 milliseconds (Holding Potential was-70 mV) from -90 mV to +60 mV (10 mV increments) in voltage clamp mode. Voltage gated delay rectifier K^+^ currents and Ca^2+^/Na^+^ inward currents were recorded to neural like cells (Fig .3). The voltage-current relationship curve showed inward currents activated at -30 mV and their maximum currents recorded at -20 mV. We observed in the exposure of K+ free and barium chloride solutions outward currents decreased and inward currents better seen on the 11^th^ and 20^th^ day (unpaired t test, P<0.05, Fig .3B). In addition, Gene expressions of Na^+^/Ca^2+^ α-subunit ion channels examined by realtime reverse transcriptase-polymerase chain reaction (qRT-PCR) technique. Expressions of the N type voltage gated calcium channel (Ca_v_2.2), L type voltage gated calcium channel (Ca_v_1.4), voltage gated Sodium channels (Na_v_1.1 and Na_v_1.2) significantly increased in the motor neuron stage (stage 5, unpaired t test, P<0.05, Fig .3).

## Discussion

In the present research, we intended to differentiate hESCs into motor neuron like cells by using an efficient and cost-effective small molecules cocktail. Our results suggested application of triplex small molecules purmorphamine, A8301 and XAV939 impressed signaling pathways towards neural differentiation, and therefore, neural progenitor cells appeared 18 days after neural induction. Increased expression of neural progenitor markers NESTIN, SOX1, and PAX6 confirmed directed activation of signaling pathways by small molecules cocktail. Previous researches showed dorsomorphin had greater effect on neural induction (7). These studies reported the combination of BMP and TFGβ inhibitors have improved the ability to direct hESCs into a neural lineage. Also in the previous study SB43152 applied for inhibiting TFGβ signaling pathway for neural induction and showed effective result (8).

In the present research with dorsomorphin, we applied A8301 as an agonist of SB43152 and inhibitor of TFGβ signaling pathway, along with XAV939 as a betacatenin- mediated transcription inhibitor of the selective Wnt pathway for neural differentiation. Liu and Zhang (11) reported that XAV939 was a Wnt inhibitor which enhanced neuron tube formation. According to previous studies, early neural induction required the presence of bFGF as an effective growth factor for differentiation of cholinergic neurons (12). bFGF was not applied for direct motor neuron differentiation from hESCs. Prolonged exposure to bFGF supported differentiation towards rostral cells, but disturbed regulation of dorsalventral identity (12, 13). In present research triplex small molecules cocktail application showed effective and affordable neural differentiation compared to prior study (13). It seems that application of dual inhibition of BMP and TGF-β signaling pathways increased efficiency of neural differentiation.

Motor neuron differentiation protocols in past researches have shown the importance of RA. Our results showed hESC-derived neuron cells expressed specific motor neuron proteins (HB9 and ISL1). Thus, accompaniment of RA and purmorphamine induced neural ectoderm structure into motor neuron phenotype. In the prior research according to HB9/ISL1 expression ratio motor neuron generation efficiency was 14% (14) whereas the efficiency of motor neuron generation in the present research enhanced to 39%. Therefore it seems that application of triplex small molecules purmorphamine, A8301 and XAV939 promoted neural differentiation effectively.

A long with cellular and molecular assessments patch clamp recording data showed existence of inwardly and outwardly voltage gated channels in hESC-derived motor neurons. Based on ion channel kinetics it seems that outwardly and inwardly currents caused by voltage-gated K^+^ and Ca^2+^/Na^+^ channels. The otherwise current clamp records suggested that hESC-derived motor neurons were generally immature because they could generate only single action potentials and unable to fire repetitive action potential (15). According to past researches 21 days were not sufficient for ion channel kinetic development therefore in term of patch clamp recording data the cells were not functionally capable to generate repetitive action potential (15, 16). There was a contradiction in observing rebound action potential in rarely cells. Pharmacological assessment in past studies showed transient K+ current and L type Ca^2+^ currents have affected the formation of rebound action potential in mature neurons. Therefore it seems that very low number of the cells was mature and showed neural higher function (16).

## Conclusion

Effective and affordable triplex small molecules cocktail can replace by expensive morphogen factors. However, there is still a need for improvement of differentiation protocols to accelerate maturation and improve cell function. According to cellular and molecular results, cellular properties of differentiated neurons are not consistent with physiological properties. We have concluded that the neural properties needed additional time to reach maturity. It seems that the cells should be more developed for electrophysiological properties on the basis of this protocol.
